# An mHealth Intervention to Improve Young Gay and Bisexual Men’s Sexual, Behavioral, and Mental Health in a Structurally Stigmatizing National Context

**DOI:** 10.2196/mhealth.9283

**Published:** 2018-11-14

**Authors:** Corina Leluțiu-Weinberger, Monica Manu, Florentina Ionescu, Bogdan Dogaru, Tudor Kovacs, Cristian Dorobănțescu, Mioara Predescu, Anthony Surace, John E Pachankis

**Affiliations:** 1 François-Xavier Bagnoud Center School of Nursing Rutgers Biomedical and Health Sciences Newark, NJ United States; 2 Insight Bucharest Romania; 3 Population Services International Romania Bucharest Romania; 4 Data Center Solutions Bucharest Romania; 5 National Institute of Infectious Diseases “Prof. Dr. Matei Balș” Bucharest Romania; 6 Department of Behavioral and Social Sciences School of Public Health Brown University Providence, RI United States; 7 Social and Behavioral Sciences Yale School of Public Health New Haven, CT United States

**Keywords:** alcohol use, young gay and bisexual men, HIV risk, mental health, mHealth intervention

## Abstract

**Background:**

Young gay and bisexual men (YGBM) in some Eastern European countries, such as Romania, face high stigma and discrimination, including in health care. Increasing HIV transmission is a concern given inadequate prevention, travel to high-prevalence countries, and popularity of sexual networking technologies.

**Objective:**

This study aimed to adapt and pilot test, in Romania, a preliminarily efficacious mobile health (mHealth) HIV-prevention intervention, created in the United States, to reduce HIV risk among YGBM.

**Methods:**

After an intervention formative phase, we enrolled 43 YGBM, mean age 23.2 (SD 3.6) years, who reported condomless sex with a male partner and at least 5 days of heavy drinking in the past 3 months. These YGBM completed up to eight 60-minute text-based counseling sessions grounded in motivational interviewing and cognitive behavioral skills training with trained counselors on a private study mobile platform. We conducted one-group pre-post intervention assessments of sexual (eg, HIV-risk behavior), behavioral (eg, alcohol use), and mental health (eg, depression) outcomes to evaluate the intervention impact.

**Results:**

From baseline to follow-up, participants reported significant (1) increases in HIV-related knowledge (mean 4.6 vs mean 4.8; *P*=.001) and recent HIV testing (mean 2.8 vs mean 3.3; *P*=.05); (2) reductions in the number of days of heavy alcohol consumption (mean 12.8 vs mean 6.9; *P*=.005), and (3) increases in the self-efficacy of condom use (mean 3.3 vs mean 4.0; *P*=.01). Participants reported significant reductions in anxiety (mean 1.4 vs mean 1.0; *P*=.02) and depression (mean 1.5 vs mean 1.0; *P*=.003). The intervention yielded high acceptability and feasibility: 86% (38/44) of participants who began the intervention completed the minimum dose of 5 sessions, with an average of 7.1 sessions completed; evaluation interviews indicated that participation was rewarding and an “eye-opener” about HIV risk reduction, healthy identity development, and partner communication.

**Conclusions:**

This first mHealth HIV risk-reduction pilot intervention for YGBM in Eastern Europe indicates preliminary efficacy and strong acceptability and feasibility. This mobile prevention tool lends itself to broad dissemination across various similar settings pending future efficacy testing in a large trial, especially in contexts where stigma keeps YGBM out of reach of affirmative health interventions.

## Introduction

HIV transmission among gay and bisexual men (GBM) is an increasing concern in Central and Eastern European countries, such as Romania, where previously reported low HIV rates are increasing [1,2]. GBM are a relatively hidden [3], yet highly vulnerable emerging HIV risk group in Romania, a country with one of the highest numbers of unrecognized HIV/AIDS cases in that region [4,5]. Currently, HIV transmission is largely driven by male-to-male sexual contact [5-7]. The Joint United Nations Programme on HIV/AIDS reports that HIV cases among GBM in Romania have nearly doubled from 8% in 2009 to 14% in 2011; however, this critical risk group has been suboptimally reached by prevention efforts, with substandard national attention dedicated to GBM health [6]. Furthermore, this group’s vulnerability has increased due to frequent travel to high-prevalence countries after Romania’s entry in the European Union in 2001 and the advent of sexual networking technologies.

Commensurate preventive resources are needed to counteract the increasing HIV incidence among Romanian young GBM (YGBM) [4,6,8-12]. However, this group faces some of the highest stigma and discrimination in Europe [12,13], including in health care [3], where GBM-specific expertise is limited. Consequently, Romanian YGBM often conceal their sexual orientation [5,14], have low rates of HIV testing [1,2], and often do not disclose their sexual behavior to providers, all of which likely lead to the rising HIV epidemic [15]. Sexual orientation stigma and subsequent discrimination have been shown to be associated with increased vulnerability [10,16], including in relation to HIV transmission, such as condomless sex (in the absence of preexposure prophylaxis), substance and alcohol use, poor mental health, and suboptimal HIV testing [17-21], which combine to form a syndemic (ie, synergistic epidemic) that further exacerbates HIV risk [21-35]. Accurate psychiatric data are not available for Romanian YGBM due to identity concealment motivated by fear and suboptimal medical record tracking [3,36]. However, nonprobability surveys and interviews suggest that Romanian YGBM report high levels of depression and alcohol abuse [37], which has been shown by numerous cross-European studies to be linked to exposure to stigma and discrimination [38-42]. Furthermore, excessive alcohol use, which is normative in Romania [42], has been shown to co-occur with sexual risk behavior [21,28,29]; by addressing mental and behavioral health, sexual risk is concomitantly addressed [26,43-45]. Prevention interventions that address HIV-related syndemics associated with stigma against YGBM are much needed in countries such as Romania.

Culturally sensitive adaptations of HIV-preventive interventions developed in other countries could provide a solution to the largely unaddressed health needs of YGBM in this region. Yet, given the lack of brick-and-mortar venues providing affirming health care for YGBM in Romania, alternative intervention delivery methods are required. Mobile health (mHealth) represents one such tool with capacity for reaching otherwise hidden populations [46]. mHealth possesses particular promise for YGBM, given their preference for Web-based sexual health support [46,47], the pervasiveness of mobile technologies throughout Romania [48-50], and the ability of mHealth technologies to bypass barriers to health care for marginalized populations by bringing prevention “to their pockets” [51].

Motivated by the lack of programming for HIV prevention in Romania, where the HIV epidemic is rapidly increasing and YGBM face difficulties accessing on-the-ground services out of fear of stigmatization, we introduced the first mobile program aimed at reducing the HIV risk among this vulnerable and underserved group. As such, this study was designed to test whether an mHealth HIV-prevention intervention would be feasible, acceptable, and preliminarily efficacious in supporting the unique HIV-prevention and related health needs of YGBM in Romania. Originally developed and successfully pilot-tested in the United States using a one-group pre-post intervention design [52], this intervention was adapted by our US-Romanian team to the local cultural context.

The present intervention is distinct from the original in several ways. First, the original intervention involved an internet (ie, Facebook)-based HIV-prevention intervention with YGBM in New York City, which the majority of participants completed via personal computer, and was not a mobile-based intervention. This study developed and tested a *mobile-based* HIV-prevention intervention in Romanian YGBM, which all participants completed via mobile devices, defined as any device that can be portable such as cellular phones, tablets, or laptops. Second, the original intervention focused on reducing recreational drug use, given the normative nature of drug use and its associations with HIV risk among the gay or bisexual population in New York City [53-57]. The current intervention focused on reducing alcohol abuse, given the normative nature of alcohol abuse and its associations with HIV risk among the gay and bisexual population in Romania [37,58]. Third, the interventions are distinct in that they were delivered in entirely different structural contexts; therefore, the Romanian study addressed structural stigma as a determinant of HIV risk and alcohol abuse. Despite these distinctions, the two interventions share certain features, namely their theoretical framework; both utilized motivational interviewing (MI) and cognitive behavioral skills training (CBST). This paper describes the intervention’s adaptation, pilot implementation, and preliminary efficacy results using a sample of Romanian YGBM with the goal of providing an efficient prevention tool that resonates with and is capable of reaching this largely hidden high-risk population.

## Methods

### Participants and Procedures

Participants were recruited between September 2015 and July 2016 through ads posted on GBM-specific Facebook sites and groups and social sexual networking apps (eg, Grindr and Planet Romeo), word of mouth, and fliers distributed in the main lesbian, gay, bisexual, and transgender (LGBT) nightclub in Bucharest, the capital of Romania. While most recruitment took place in Bucharest, we were able to reach men in other towns of Romania through word of mouth and Facebook. The ads contained a link to a 5-minute Web-based screener assessing sexual behavior, alcohol use, HIV status, and HIV testing patterns. In order to qualify for both formative and intervention phases of the project, men had to (1) be aged between 16 and 29 years; (2) self-report an HIV-negative or unknown status; (3) have had at least one condomless anal sex act with a male partner in the past 3 months; and have either (4) at least 5 heavy drinking days (at least 5 standard drinks on one occasion) or (5) at least one condomless anal sex act under the influence of alcohol in the past 3 months. These criteria are based on established associations between alcohol use and condomless sex [55,59-65]. Those eligible were given the option to enter their contact information in a separate survey for the study coordinator to contact them by phone for enrollment. Each participant received a link to an informed consent document. The study coordinator answered participants’ questions and verified their identity and age through an electronic copy of a government-issued identification card sent by the participant. After consent, each participant received a secure link to a 30-45-minute Web-based baseline assessment. Participants completed the same survey approximately 3 months postbaseline, as an immediate postintervention follow-up assessment (given that participants took between 2 and 3 months to complete their 8 weekly intervention sessions). The baseline and follow-up assessments were compensated with the equivalent of US $20 and US $40, respectively, provided to participants in gift cards at the end of their participation. Each completed session was compensated with the equivalent of US $10, allocated as above. Consent, assessments, and counseling sessions were conducted in Romanian, while all translation was overseen by one of the investigators who is a native Romanian speaker fluent in English. As the other investigator did not speak Romanian, whenever participants spoke fluent English (97% of the time), formative and evaluation interviews were conducted in English, given the high English language proficiency in Romania. When participants did not speak English, the bilingual investigator conducted interviews in Romanian, which she also translated into English for the other investigator (who was present for all interviews) and back into Romanian for the participants. The Romanian study team spoke English fluently. All procedures were approved by the Human Research Protections Program at Hunter College of the City University of New York and Yale University, USA.

### The Intervention

#### Adaptation

The intervention, called “Despre Mine. Despre Noi.” (DMDN), translated into English as “About Me. About Us.,” was adapted for the cultural context of Romania from a US-based Web-based intervention that showed preliminary efficacy for reducing sexual risk and substance use while delivered on Facebook by trained counselors to YGBM at risk for HIV [52]. For the intervention to resonate with the Romanian GBM community, the 2 principal investigators conducted a focus group in December 2013 with YGBM (n=8) as an initial *general* needs assessment. In December 2014, the investigators returned to Romania to conduct 60-90-minute individual interviews with a new set of YGBM (n=22), infectious disease physicians from the National Institute of Infectious Diseases (n=3), and LGBT community advocates (n=3), as a more comprehensive *intervention* needs assessment. The focus group and interviews were audiorecorded and coded for pertinent themes related to the biopsychosocial context of HIV risk and prevention needs for YGBM in Romania and ideal delivery modalities for this unique context. Specifically, the themes assessed the unique sexual (eg, HIV risk, condom use patterns, and partner communication), behavioral (eg, contexts and motivations for excessive alcohol use), and mental (eg, depression, social isolation, relationship strain, coming out) health needs of Romanian YGBM to inform intervention content, structure, and optimal elements for a secure mobile study platform. Once the investigators extracted relevant themes from the focus group and interviews, they modified the original intervention to incorporate these elements and met in May 2015 with 15 of the original interviewees for 60-minute individual interviews, in order to walk them through the modified intervention to gather and incorporate additional feedback. In addition, the participants navigated the mobile platform (a “mobile device” is considered to be a mobile phone, a tablet, or a laptop given their portability) and provided feedback to the technical developer who was present at all the meetings. Final content-related and technical modifications were made before intervention launch based on iterative user testing by the YGBM and study team.

#### Content

The intervention aimed to support YGBM in reducing their condomless anal sex and alcohol use and improving their mental health. The 8 counseling sessions lasted 60 minutes each and were delivered through the chat feature of our study mobile platform. The intervention was based on the principles and techniques of MI [66,67] utilized to help participants contemplate and prepare for change to reduce HIV-risk behavior. This was supplemented by CBST [68,69] and information for accessing local health-promoting resources. A description of the core intervention elements has been published previously [52]. The DMDN intervention maintained its original 8 60-minute session structure [52]. Focus group and interviews during the adaptation phase yielded the following unique elements to support Romanian YGBM in their highly stigmatizing national context, leading to increased HIV risk behavior yet offering sparse HIV prevention and information resources: (1) basic HIV/AIDS education; (2) raising awareness of the importance of HIV testing, importance of sexual health communication with partners, and health-promoting resource utilization; (3) support for sexual identity development and coming out, including offering the intervention to YGBM as young as 16 years of age (given their high familial and societal rejection); and (4) specific technical features.

#### Technical Platform

Given the mobile nature of the intervention, we gave participants the option to use any mobile device they preferred, including mobile phones, laptops, and tablets. DMDN is a mobile intervention, given that it is portable and can be accessed from anywhere by participants. A local software developer constructed and maintained the mobile site used to deliver the intervention and provide HIV-related information or resources to participants. The site had the following four domains: (1) a chat page with interfaces to chat with one’s counselor; (2) a resource page containing HIV/AIDS-related information and links to relevant resources (eg, LGBT-affirmative testing clinics, European Centers for Disease Control fact sheets, thebody.com); (3) a behavioral and affective tracking tool where participants entered weekly reports of their number of sex partners and condomless anal sex acts with male partners, alcohol consumption, and negative and positive affect (eg, emotions such as “angry” or “happy”); and (4) account settings. Participants used the behavioral and affective tracking tool as a visual gauge of their behavior over time, which was reviewed in sessions with counselors to motivate behavior change and guide their discussions. The tracking tool was used on a weekly basis for the duration of the intervention.

Once participants completed their baseline assessment, the study coordinator notified their assigned counselor, who scheduled the first session. On the set day and time, the counselor and participant logged into the mHealth study platform and commenced the sessions.

### Counselor Training and Supervision

Prior to intervention launch, the 2 US-based investigators held a 2-day training in May 2015 in Bucharest with 3 Master’s level Romanian counselors—2 psychologists and 1 LGBT community advocate with experience in MI and HIV-prevention interventions. A training manual, written originally for the US-based intervention and adapted (by including the aforementioned elements) for the Romanian context by the investigators, was used. The training included both didactic and experiential components, a review of principles and techniques of MI and CBST, the unique nature of text-based therapeutic communication, and vignettes and exercises drawn from the investigators’ previous interventions. Next, the counselors practiced each session via text-based communication, each taking turns being a mock participant and counselor and receiving remote video supervision from the clinical psychologist investigator. Once the intervention began, the counselors saved the transcripts from each session, translated them into English, and received video-based supervision from the clinical psychologist investigator, guided by the intervention manual, on a weekly basis for the duration of the study.

### Measures

#### Demographics

Demographic items assessed age, gender, sexual orientation, level of education, current employment, income, and relationship status.

#### Sexual Behavior

To assess *HIV-risk behavior*, we asked participants how often they had anal and vaginal, insertive and receptive, sex with and without condoms with various sex partners (steady male or female, casual male or female, and transgender male or female) over the past 3 months. *Condom use self-efficacy* was assessed using a 13-item scale [70] (alpha=.98). An item example is “How confident are you that you could avoid having sex without a condom when you really need affection?,” measured using a 5-point Likert scale ranging from 1 “not at all confident” to 5 “extremely confident.”

#### HIV/AIDS Knowledge and Information Seeking

To assess *HIV/AIDS knowledge*, we used a 13-item scale (alpha=.71) based on the recommendations of the United Nations General Assembly Special Session on HIV/AIDS and the European Centers for Disease Control [71,72] with modifications implemented in previous survey research with European, including Romanian, GBM [5]. An example item is “If someone becomes infected with HIV it may take several weeks before it can be detected in a test.” Response options ranged from 1 “I do not believe this” to 5 “I already knew this.” Four items (alpha=.71) [5] were used to assess *HIV/AIDS information-seeking*, with an item example being “When was the last time you actively looked for information about HIV or STIs on the internet?” Response options ranged from 1 “within the last 12 months” to 5 “within the last 24 hours.” *Recency of HIV testing* was assessed by asking “When was your last HIV test?,” with response options ranging from 1 “never” to 5 “3 months or less” [73,74].

#### Alcohol Use

We asked participants to report the number of days in the past 3 months on which they had had at least 5 standard drinks, considered to be “heavy drinking days” [52,55,75-77]. In addition, participants were asked to report the number of alcoholic drinks consumed on a typical drinking day [78,79]. We also measured *self-efficacy to reduce alcohol use* with a 15-item scale (alpha=.97) [80]. An item example is “I would be able to resist the urge to use alcohol if I were out with friends and they kept suggesting we go somewhere to drink.” Response options ranged from 1 “not at all” to 5 “completely.”

#### Psychosocial Outcomes

*Depression and anxiety symptoms* were assessed with the 12-item Brief Symptom Inventory Scale [81] depression and anxiety subscales (alpha=.92 and alpha=.93, respectively). Participants were asked on a Likert scale ranging from 0 “not at all” to 4 “extremely,” how intensely they experienced a variety of symptoms in the previous 7 days, such as “nervousness or shakiness inside” or “feelings of worthlessness.”

#### Training Acceptability

We conducted 15 postintervention interviews with randomly selected participants (15/43, 35%) to gather feedback on their experiences. We randomly selected participants from 2 types of categories, those who completed all 8 intervention sessions (n=12) and those who did not (n=3), in order to obtain input on the intervention experience representative of both groups for future intervention improvements. We only selected 15 participants to interview because based on our interviews for the formative phase of this and other interventions, theme saturation is reached once 12-15 participants are interviewed. We similarly reached theme saturation after interviewing 15 participants in this study. The interview assessed (1) the feasibility of using mobile technologies to receive counseling (eg, “How did it feel for you to communicate via text with your counselor about sexual behavior and everything else you discussed?”); (2) general program perceptions (eg, “What did you like the most/least about this experience and why?”; (3) perceived impact of participation (eg, “How was your health affected by the program?”); (4) the quality of the therapeutic relationship (eg, “Did you feel you could trust your counselor and if so, why? Or why not?”; and (5) evaluation of the mobile platform (eg, “Tell us about what you would keep and/or change about the color scheme, images, text, interface, sections, and tabs.” The investigators kept detailed interview notes to measure the intervention’s acceptability.

### Pre-Post Intervention Analyses

Descriptive statistics were obtained for demographics. The number of sexual acts with and without a condom was calculated by summing across all pertinent items. Prior to computing mean scale scores, necessary items were reverse-coded. We conducted paired-samples *t* tests to detect possible changes from the baseline to the follow-up in our outcomes. The significance level was set at *P*<.05. All analyses were conducted using SPSS 23 (IBM Corp, Armonk, NY, USA) [82].

## Results

### Participant Enrollment and Intervention Completion Rates

**Figure 1:**
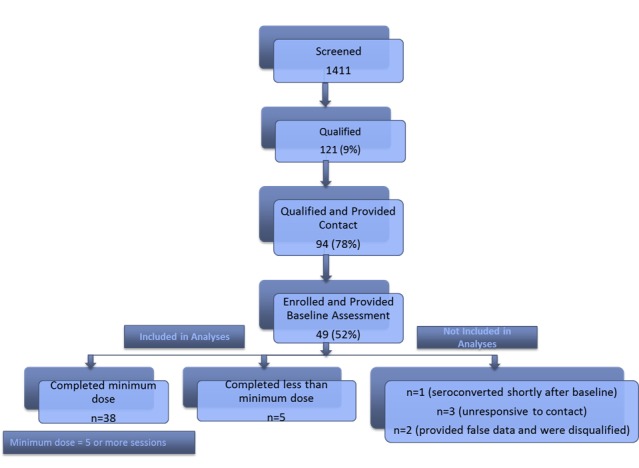
Consolidated Standards of Reporting Trials flow diagram.

Participants accessed the eligibility screen through apps (635/1411, 45.0%), Facebook (579/1411, 41.0%), and word of mouth or in the local gay nightclub (197/1411, 13.9%). Nearly half (607/1411, 43.0%) of those who screened (n=1411) were from Bucharest. Of the 1411 individuals, 8.6% (121/1411) met the eligibility criteria. Some of the men who screened omitted reporting on sexual behavior (likely due to its stigmatized nature in Romania), which automatically eliminated them from being assessed for potential risk and therefore participation. Due to these missing data, it was not possible to determine whether or not a large proportion of the screening sample did not present risk or simply did not report it. As such, 39.9% (564/1411) of the men who screened did not meet the sex risk criteria and 22.9% (324/1411) did not report on the sex risk criteria; 16.9% (239/1411) did not use alcohol in the past 3 months; 23.9% (338/1411) did not report heavy drinking in the past 3 months; 7.9% (112/1411) were HIV-positive; and 2.9% (42/1411) did not report their status. Given that the eligibility was determined on the basis of a combination of risky sexual behavior, excessive alcohol use, and HIV status, missing data and the need for overlap across these 3 criteria yielded a relatively small final eligible sample. Of the 121 eligible men, 77.6% (94/121) provided contact information, and 52% (49/94) of these enrolled and completed the baseline survey. Of these, 90% (44/49) began the intervention and 86% (38/44) of those who began the intervention completed the minimum dose of 5 sessions. The minimum dose of 5 sessions was established based on the fact that the first 5 sessions convey the core elements of the intervention, while the remaining sessions are dedicated to behavioral skills practice, consolidating treatment gains, and establishing a plan to maintain progress. A high proportion (38/44, 86%) of our participants received the essential intervention elements. The session completion breakdown is as follows: 82% (36/44) completed all 8 sessions, 2% (1/44) completed 7 sessions, 2% (1/44) completed 6 sessions, 4% (2/44) completed 4 sessions, and 9% (4/44) completed 1 session. A total of 88% (43/49) participants completed both the baseline and follow-up questionnaires and constituted the analytic sample. The average number of sessions completed was 7.1; each session lasted 60 minutes. The average length between sessions was 1.5 weeks, with the goal of completing one session per week (inevitable scheduling conflicts arose). Of note, 6 enrolled participants were excluded from the final analyses—1 who seroconverted shortly after the baseline, 3 who were unresponsive to our contact efforts, and 2 who provided false data and were disqualified. Figure 1 provides the Consolidated Standards of Reporting Trials flow diagram of participants from screening to data analyses.

### Demographic Distribution

Table 1 describes the baseline sample characteristics. The majority (35/43, 81%) were aged ≤24 years (mean 23.39 years, SD 3.6; range 17-29 years) and gay (30/43, 70%). Nearly half of the participants had at least a college degree (18/43, 42%) and were working full time (20/43, 47%). Nearly one-quarter (10/43, 23%) were students. The majority (26/43, 60%) of the sample indicated being single.

### Sexual Behavior and Alcohol Use

Table 2 provides comparisons of participants’ sexual behavior, alcohol use, and condom use self-efficacy pre-post intervention. Condom use self-efficacy increased significantly from baseline to follow-up: mean 3.3 (SD 1.0) versus mean 4.0 (SD 1.6), *P*=.01. Participants’ number of sex acts without a condom decreased (mean 15.3, SD 13.8 vs mean 14.9, SD 17.4, *P*=.90) and number of sex acts with a condom increased (mean 6.3, SD 7.3 vs mean 8.6, SD 9.8, *P*=.12) from baseline to follow-up, although not significantly.

Participants reported significant reductions in the number of heavy drinking days (mean 12.8, SD 19.3 vs mean 6.9, SD 10.2, *P*=.005) and drinks consumed on a typical drinking day (mean 2.2, SD 0.9 vs mean 1.9, SD 0.8, *P*=.01). In addition, they reported significant increases in self-efficacy to avoid alcohol consumption (mean 3.5, SD 1.7 vs mean 4.2, SD 1.4, *P*=.02).

### HIV Testing and Knowledge

Table 2 also illustrates participants’ changes in HIV testing and knowledge. Participants reported more recent HIV testing at follow-up than at baseline: mean 2.8 (SD 1.5) versus mean 3.3 (SD 1.7), *P*=.05. Specifically, 8 participants reported testing in the previous 3 months at baseline, and 18 participants reported testing in the previous 3 months postintervention, therefore having tested during the intervention period (*P*=.05). Furthermore, participants reported significant increases from baseline to follow-up in their HIV/AIDS-related knowledge (mean 4.6, SD 0.3 vs mean 4.8, SD 0.2, *P*=.001) and information seeking (mean 3.8, SD 1.0 vs mean 4.4, SD 1.1, *P*=.001).

### Psychosocial Outcomes

Table 2 presents changes in psychosocial outcomes. Participants reported significant decreases in both depression and anxiety symptoms from baseline to follow-up (*P*=.003 and *P*=.02, respectively).

### Intervention Feasibility and Acceptability

First, we examined session completion and reasons for not completing all 8 sessions, as indicators of intervention acceptability. Minimum dose session completion was high at 86% (38/44), with an average of 7.1 completed sessions, indicating high acceptability and feasibility. In addition, we asked participants who did not complete all 8 sessions to provide an explanation for that, and the only stated reason provided was having “too busy” of a schedule, which was unrelated to the intervention.

Second, the investigators distilled the most salient themes reflective of participants’ experiences in this pilot intervention. These themes are described below, and interview quotations appear in Textbox 1.

#### Feasibility of Using Mobile Technologies to Receive Counseling, General Program, and Counseling Relationship Perceptions

The majority completed their sessions from laptops. Communication about all sensitive topics and relevant issues via text was perceived as “natural,” and miscommunication was not normative. Several participants mentioned that they preferred communication in writing over face-to-face communication because writing provided them with more opportunities to think thoroughly about what they wanted to express. The counseling relationship was characterized as honest and trustworthy, and this was most participants’ first therapeutic encounter, which they characterized as highly positive.

#### Most Important Lessons Learned

Participants deemed the intervention as instrumental in helping them to achieve personal goals. The most important lessons learned fell under the umbrellas of HIV prevention (sexual risk and alcohol use) and psychological well-being (self-evaluation and partner dynamics).

### HIV Prevention: Sexual Risk and Alcohol Use

Participants indicated having gained significant HIV knowledge, including transmission facts, the importance of alcohol reduction, condom use, and testing (Textbox 1).

### Psychosocial Well-Being

Participants frequently described improvements in their self-awareness, gay identity, relationships, and well-being as they worked with their counselor to establish personal health goals and learn skills to achieve them.

### Intervention Structure and the Mobile Platform

The majority of participants recommended a 10-week program, rather than an 8-week program. One participant subsequently sought counseling outside of the study. Others were less eager to do so because they preferred a texting format over a face-to-face format. In addition, participants suggested flexibility around the 60-minute session to include an additional 10-15 minutes when needed. The platform was found to be easily navigable and intuitive, although a few suggested more vivid color schemes and links to relevant videos.

**Table 1:** Participant demographic characteristics (n=43).

Characteristics		n (%)
**Age (years)**		
	17-24	35 (81)
	25-29	8 (19)
**Sexual identity**		
	Gay	30 (70)
	Bisexual	12 (28)
	Uncertain	1 (2)
**Education**		
	High school or less	15 (35)
	College student	10 (23)
	College degree	13 (30)
	Graduate degree	5 (12)
**Employment**		
	Full time	20 (47)
	Part-time	1 (2)
	Self-employed	4 (9)
	Unemployed	3 (7)
	Other (students or volunteers)	15 (35)
**Relationship status**		
	Partnered	17 (40)
	Single	26 (60)
**Level of religiosity**		
	None to low	25 (58)
	Moderate to high	18 (42)
**High school location**		
	Small town	22 (51)
	Medium to large town	21 (49)
**Geographical region of residence**		
	South	27 (62)
	North East	8 (18)
	Central	6 (15)
	West	2 (5)

**Table 2:** Reported HIV-related behavior, alcohol use, HIV/AIDS knowledge, and mental health outcomes pre-post intervention.

Outcomes^a^		Baseline, mean (SD)	Follow-up, mean (SD)	Test statistic
**Sexual behavior**				
	Sex acts without a condom	15.3 (13.8)	14.9 (17.4)	Not significant
	Sex acts with a condom	6.3 (7.3)	8.6 (9.8)	*t*_19_=−1.6^b^
	Condom use self-efficacy	3.3 (1.0)	4.0 (1.6)	*t*_42_=−2.6^c^
**HIV-related health self-efficacy patterns**				
	HIV/AIDS knowledge	4.6 (0.3)	4.8 (0.2)	*t*_42_=−3.4^d^
	HIV/AIDS information seeking	3.8 (1.0)	4.4 (1.1)	*t*_42_=−3.5^d^
	HIV testing recency	2.8 (1.5)	3.3 (1.7)	*t*_41_=–2.05^e^
**Alcohol use**				
	Number of heavy drinking days	12.8 (19.3)	6.9 (10.2)	*t*_34_=3.0^f^
	Number of drinks on a typical drinking day	2.2 (0.9)	1.9 (0.8)	*t*_39_=2.6^c^
	Self-efficacy in reducing drinking	3.5 (1.7)	4.2 (1.4)	*t*_41_=−2.4^g^
**Mental health**				
	Anxiety	1.4 (1.0)	1.0 (0.9)	*t*_42_=2.45^g^
	Depression	1.5 (1.1)	1.0 (0.9)	*t*_42_=3.26^h^

^a^Not every respondent provided answers to each question and not every question was applicable to all participants (eg, main or casual partner sexual behavior); therefore, the n varies across certain outcomes.

^b^*P*=.12.

^c^*P*=.01.

^d^*P*=.001.

^e^*P*=.05.

^f^*P*=.005.

^g^*P*=.02.

^h^*P*=.003.

**Intervention feasibility and acceptability (n=15).**

**Feasibility of Text-Based Counseling**

*Even talking with someone about things you cannot talk to your friends or family about is a big plus, it helps you for the first time, it’s private; she also highlighted the strong parts of me, every time we searched for something, and at the end of the session we had a result.* [Age 22 years]

*We had wonderful talks, seriously. I started the program very down and she helped. She managed to help me see the glass half full again and not to forget certain things [such as] how not to be clouded in negative emotions. She helped me clarify [the] type of things I [should] change.* [Age 27 years]

*I thought that I have a friend I was talking to, even though I didn’t know her…This was the first time for me when I talked with a stranger very openly.* [Age 20 years]

*It felt safe in the program because I could be honest.* [Age 20 years]

*The counselor was very open-minded, nonjudgmental. It was cathartic…We had a great relationship…Excellent communication with her.* [Age 27 years]

*I felt free to talk, I was home and not in a strange office. It was easy, no misunderstandings.* [Age 20 years]

**Most Important Lessons Learned**

1. HIV Prevention: Sexual Risk and Alcohol Use

*[I learned] about how to prevent the transmission of HIV or how responsible I am about it.* [Age 20 years]

*[We] talked about new partner situations and [when] to ask for condoms.* [Age 24 years]

*She made me realize that I don’t have to seek alcohol when I feel bad, alcohol was not a solution;…even talking about it, it makes you think about it, if you don’t, you don’t realize you’re drinking like that.* [Age 22 years]

*She helped me explore the cause of my drinking, [for example], self-esteem, why do I drink?* [Age 27 years]

*I don’t use a lot [of alcohol], but through my conversations I realized that sometimes I can exaggerate and this is not a good thing. [She] helped me to become more aware of my alcohol use.* [Age 20 years]

*[We discussed] alcohol and safe sex, talked about many things that helped and I realized [my] strong points that can help in difficult situations…[I] found my strengths and the program helped me understand myself better.* [Age 24 years]

*The most important thing that I’ve learned is about the importance of protected sex. Just talking has made me appreciate how important it is to have a rubber on.* [Age 27 years]

*Of course it helped to learn more about STIs.…The primary subject [was] how important it is to protect yourself in any situation and about health to be prudent about partners you may have and alcohol use.* [Age 20 years]

2. Psychosocial Well-Being

*Most important thing I learned was to be more confident and feel better about myself.* [Age 24]

*I know myself better compared to before;…I can be more calm or I [can] see things from all perspectives.* [Age 20 years]

*The program boosted my confidence [and] after this [study], I came out to my sister, cousin, two coworkers, friends.* [Age 25 years]

*We talked about being gay…, also related to finding a job. We sometimes would stray from talking about alcohol and sex to talk about problems at home, having too much sex.* [Age 27 years]

*It was quite interesting, a pleasant experience. I had more to gain than just talking about HIV and alcohol. I started to realize more things about myself: I don’t have much trust in myself, but talking with someone and listening to myself I started to think that I can trust myself… I had a different state of mind after eight sessions, I felt better…to be more prudent and more careful with myself in every way.* [Age 20 years]

## Discussion

### Principal Findings

This study adapts and preliminary tests an HIV-prevention intervention for YGBM [52] in Romania, a country with a high degree of homophobic stigma, increasing HIV prevalence among GBM, and scarce GBM-affirmative resources [3]. After adapting a US-based intervention in close consultation with Romanian key informants, we tested it for preliminary efficacy with 43 at-risk YGBM. Comparison of outcomes before and after participation in the 8 MI-based and CBST-based mobile counseling sessions indicated improvements in sexual (eg, HIV knowledge and testing), behavioral (eg, alcohol use and alcohol and condom use self-efficacy), and mental (eg, depression and anxiety symptoms) health. This intervention shows potential promise for improving the full spectrum of psychosocial health risks that disproportionately affect this vulnerable and underserved group.

Although trends toward reduced condomless sex and increased condom use were not significant in our relatively small pilot sample, participants evidenced markedly increased condom use self-efficacy postintervention. As self-efficacy is a precursor to behavioral change [83], longer follow-ups with larger samples might reveal that this intervention can impact condom use, as mediated by improvements in self-efficacy. Furthermore, we found significant increases in HIV/AIDS-related knowledge, information seeking, and testing, suggesting that DMDN can improve HIV transmission risk prevention.

Both alcohol consumption and self-efficacy for reducing it evidenced marked changes in the intended direction. In addition, participants benefited from markedly improved mental health (eg, reduced depression and anxiety). These gains are salient in a country where alcohol consumption is among the highest in Europe [84] and where LGBT individuals face pervasive stigma deleterious to mental health [3]. Furthermore, alcohol use and mental health difficulties are associated with sexual risk, forming a syndemic (ie, synergistic epidemic) that threatens the health of YGBM [23,55,64,65,85].

The intervention was found to be highly acceptable to the Romanian YGBM community. Postintervention interviews with over one-third of the sample pointed to participants’ newfound sense of self-worth and openness toward future counseling. For most participants, this intervention provided a first outlet for exploring their gay or bisexual identity, articulating and setting sexual health goals, and learning to communicate with partners about risk. In addition, participants reported increased awareness of HIV risk and preventive behaviors (eg, testing). Thus, the intervention was embraced as a novel utility to YGBM health.

Awaiting this intervention’s efficacy testing via a large randomized controlled trial, it is worth reflecting on its potential for sustainability and dissemination, given that it entails 8 60-minute sessions delivered by trained counselors. The very high-risk nature of that target population for which it is designed—at risk of HIV infection, alcohol abuse, and depression—necessitates a commensurately intense intervention. Whereas a briefer intervention might be warranted for risk reduction in the general population, it is likely unsuitable for syndemically affected sexual minority groups, and 8 sessions fall within the standard range for interventions targeting multiple outcomes in high-risk GBM [43]. Furthermore, structural barriers to implementing a relatively intensive intervention are modifiable to maximize dissemination and sustainability potential. Specifically, in related research [86], we recently trained 180 Romanian mental health providers in GBM-affirmative treatment and have created a mobile platform to provide them with ongoing LGBT-affirmative supervision so that our DMDN intervention, if efficacious pending rigorous testing, will persist after the study concludes. This approach provides a blueprint for sustainability and longer-lasting impact. Furthermore, hybrid individual-structural interventions may be applied concomitantly in similar settings to ameliorate the effects of stigma on individual health, and target it structurally at its source. Finally, similar interventions have included both professional and peer counselors, and regardless of the modality of intervention delivery, it is essential that adequate training be provided.

### Limitations and Future Directions

Our findings should be considered alongside several limitations, which point to future research directions. First, although >4000 individuals screened for study eligibility, only 8.6% (121/1411) qualified, of whom 77.7% (94/121) were willing to provide contact information. This is unsurprising given that communication about sexual behavior remains taboo and unsystematically addressed in Romania [87,88], which is further complicated by asking men to communicate about same-sex sexual behavior, another highly stigmatized topic. At this early stage of sexuality-related research among Romanian YGBM, hesitance to provide information to researchers is to be expected. While recruitment was effortless, future recruitment efforts might consider increasing YGBM’s comfort with providing accurate personal information, such as further emphasizing confidentiality. Second, this study did not include a control group, which should be considered for a future randomized controlled trial to validate the intervention’s efficacy in changing sexual, behavioral, and mental health outcomes. Third, a larger sample will provide sufficient power for determining whether the nonsignificant trends we recorded in sexual risk reduction are statistically and clinically meaningful. Fourth, a longer-term follow-up (eg, up to 24 months) would permit measuring the durability of intervention benefits and the potential impact of booster sessions. Fifth, future studies might consider broader geographical representation of YGBM using social media recruitment outreach campaigns. Expanded outreach beyond major cities and surrounding areas will increase the representation of YGBM who are isolated and outside the perimeter of preventive guidance, and who would likely benefit the most from this intervention. Finally, supplementing the evaluation interviews with a measure of intervention acceptability as a rating of its usefulness, ease of use, likelihood of future use, or recommendation to peers in the future would have bolstered our evaluation of this intervention. The inclusion of such scales in future intervention evaluations is warranted.

### Conclusions

This first step in addressing Romanian YGBM’s sexual, behavioral, and mental health through mHealth interventions lays the groundwork for larger trials capable of establishing the intervention’s efficacy before broad implementation. Awaiting proof of efficacy, this intervention could eventually be adopted by public health care entities and LGBT-affirmative practitioners looking to reach broader segments of the population than possible through face-to-face means. Furthermore, the DMDN intervention may lend itself to wide dissemination to GBM without access to conventional sources of health support.

## Abbreviations

CBST: cognitive behavioral skills training
DMDN: Despre Mine. Despre Noi.
GBM: gay and bisexual men
LGBT: lesbian, gay, bisexual, and transgender
MI: motivational interviewing
mHealth: mobile health
YGBM: young gay and bisexual men
